# **Anti-tumor and immunomodulatory activity of** *Ganoderma parvulum-*derived **polysaccharides**

**DOI:** 10.1042/BSR20240113

**Published:** 2025-10-13

**Authors:** Katherin Contreras-Ramirez, Xiomara López-Legarda, Jorge H. Tabares-Guevara, Juan C. Hernandez, Freimar Segura-Sánchez, Janny A. Villa-Pulgarin

**Affiliations:** 1 Grupo de Investigaciones Biomédicas UniRemington, Facultad de Ciencias de la Salud, Corporación Universitaria Remington, Medellín, Colombia; 2 Grupo de Investigación Biopolimer, Facultad de Ciencias Farmacéuticas y Alimentarias, Universidad de Antioquia UdeA, Medellín, Colombia; 3 Infettare, Facultad de Medicina. Universidad Cooperativa de Colombia, Medellín, Colombia

**Keywords:** anti-tumor activity, β-glucans, cancer immunotherapy, fungal polysaccharides, macrophages

## Abstract

Polysaccharides have gained considerable attention recently because of their anti-tumor and immunoregulatory properties. Its activity depends on the type of fungus that produces it, the extraction method, and the molecular weight. **Materials and Methods:** This study evaluated the anti-tumor and immunoregulatory properties of *Ganoderma parvulum* (GP)-derived polysaccharides. **Results:** GP crude polysaccharides inhibited mice’s lymphoma without cytotoxicity. *In vitro* studies showed that exopolysaccharides (GEPS) and intrapolysaccharides (GIPS) of *G. parvulum* inhibited the proliferation of tumor cells, but no cell death was observed. Significantly, GEPS and GIPS increased the production of nitric oxide, TNF-α, MCP-1, and an increase in IL-1β, IL-18, and Caspase-1, while NLRP3 was down-regulated. Also, it decreases the production of IL-10 and IL-6. **Conclusion:** These observations suggest that GP-derived polysaccharides may exert anti-tumor activity primarily by activating the host’s immune system via macrophage stimulation.

## Introduction

Traditional cancer treatments, including surgery, radiotherapy, chemotherapy, and immunotherapy, have recently revolutionized cancer treatment [1,2]. Despite this, few patients experience clinically effective outcomes from cancer treatment [3]. Studies have shown that the immune system can inhibit the development of many malignant tumors. Immunomodulatory compounds of natural origin have a wide adjuvant therapeutic potential in neoplastic diseases, allergies, and several chronic and infectious diseases [4,5]. Although many immunomodulators are unknown in their mechanisms of action, these cause changes in gene expression, mRNA processing, protein synthesis, transport and secretion, and cell surface protein expression, which influence the induction, maintenance, and regulation of the immune response [6].

Recently, studies on new anti-cancer treatments from mushrooms have been significantly developed, mainly because they contain a broad range of bioactive polymers such as polysaccharides [7]. Molecules such as these may adopt pathogen-associated molecular patterns and modulate macrophages and granulocyte responses to pathogens. Recent data suggest fungi polysaccharides have potential immunomodulatory and anti-tumor effects [8]. Lentinan (a 1,3-glucan) is a commonly used anti-tumor drug derived from the fruiting body of *Lentinus* edodes [9]. Macrophages and monocytes can recognize β-glucans via various receptors [10]. It has been established that dectin-1 receptors play a key role in recognizing β-glucans. Activation of dectin-1 with β-glucans can trigger cytokine release and phagocytosis [11]. Polysaccharides of *Ganoderma lucidum*, *Agaricus blazei, Lentinus edodes, Cordyceps sinensis, Cantharellus cibarius,* and *Arillaria mellea* promoted the expression of pro-inflammatory cytokines in macrophages as well as the phagocytic capacity, indicating the activation of a macrophage-mediated immune response, besides increasing the production of TNF-α, NO, ROS, IL-1β, IL-12, and IL-6 in M2-macrophages and promoted their transformation to M1-macrophages [10–12]. According to these data, macrophages can be primed with β-glucan, resulting in rapid and effective immune responses against engrafting tumor cells, possibly through macrophage-mediated anti-tumor mechanisms [13]. Polysaccharides can actively manipulate the tumor microenvironment (TME) [10,14].

Colombia has many ligninolytic fungi, but their potential uses have not yet been explored. In our previous studies, we isolated and identified wild mushrooms from tropical forests (Colombia) for submerged cultivation [15]. Their intra- and exopolysaccharides showed anti-tumor and immunomodulatory properties *in vitro [16,17]*. It was found that these polysaccharides have *in vivo* anti-tumor effects in the absence of cytotoxicity. Additionally, two water-soluble fractions of *Ganoderma parvulum* polysaccharides (intra-polysaccharide GIPS and exo-polysaccharide GEPS) were evaluated for their anti-tumor and immunostimulant properties *in vitro*. We reported that those polysaccharides significantly inhibited the proliferation of U937, EL-4, A549, and MDA-MB234 tumor cell lines in a dose-dependent manner. Besides, these GPs promoted the secretion of pro-inflammatory cytokines such as TNF-α and MCP-1. They up-regulated the expression of IL-1β, IL-18, and Caspase-1, demonstrating the activation of the macrophage towards an M1 profile, which could be related to its anti-tumor properties *in vivo*.

## Materials and methods

### Reagents

Culture Medium Dulbecco’s Modified Eagle (DMEM, D6429), Sigma-Aldrich brand (Saint Louis, MO, U.S.A.), Fetal Bovine Serum (FBS, 102–500) Macrogen brand (Bogotá, CO), antibiotics Penicillin (10,000 IU/ml) and Streptomycin (10,000 μg/ml) (30–002-Cl) CORNING brand (Arizona, U.S.A.), MTT reagent (3-(4,5-dimethyl-2-thiazolyl)-2,5-diphenyl-2H-tetrazolium bromide, 5224)) TOCRIS brand (Bristol, U.K.), LPS (lipopolysaccharides L3137) Sigma-Aldrich brand (Saint Louis, MO, U.S.A.), Griess reagent (G4410) Sigma-Aldrich brand (Saint Louis, MO, U.S.A.), Recombinant Interleukin 4 (IL-4 214–14) Peprotech brand (Cranbury, NJ, U.S.A.), BD Cytometric Bead Array Mouse Inflammation Kit (CBA 552364) BD Bioscience brand (CA, U.S.A.), TRI® reagent (TRI R2050-1-200) ZYMO RESEARCH brand (Irvine, CA, U.S.A.), Direct-zol™ RNA MiniPrep with Zymo-Spin™ IIC Columns (R2052) ZYMO RESEARCH brand (Irvine, CA, U.S.A.), iScript cDNA Synthesis Kit (1708891) Bio-Rad brand (Hercules, CA, U.S.A.), QuantinNova® PCR SYBR Green Kit (208054) QIAGEN brand (Hilden, Germany), and Purified PRIMERS KIT 50 NMOL (β-actin, TNF-α, IL-1β, IL-6, Mrc1, Arg, iNOS, Fizzy1) Macrogen brand (Bogotá, CO).

### Mice

Eight-weeks-old C57BL/6 female wildtype (WT) mice weighing 18–20 g (Charles River, Portage, MI, U.S.A.) were bred and housed at 22 ± 1°C under a 12 h light/dark cycle with food and water ad libitum and maintained under specific pathogen-free conditions at the animal facility of the Sede de Investigación Universitaria (SIU), Universidad de Antioquia (UdeA). At the end of the experiments, euthanasia was performed using an overdose of ketamine/xylazine 100/10 mg/kg intraperitoneally (IP). All experiments were approved by the corresponding Institutional Animal Care and Use Committees (Comité Institucional para el Uso y Cuidado de los Animales de Experimentación, CICUA, UdeA: minutes #102-April 14, 2016), and performed following local and international guidelines and regulations.

### Allograft mouse model

Six- to eight-week-old C57BL/6 female WT mice (*n* = 7) were inoculated subcutaneously into their right flank with 2.5 × 10^5^ EL-4 cells in 500 μl PBS. When tumors were palpable, mice were randomly assigned into cohorts of seven mice each group, receiving a daily oral administration of 100 and 200 mg/kg of polysaccharides of *G. parvulum* in PBS or an equal volume of PBS (control). The shortest and longest diameter of the tumor was measured with calipers at two-day intervals, and tumor volume (mm^3^) was calculated using the following standard formula: (the shortest diameter) 2 × (the longest diameter) × 0.5. Animal body weight and any signs of morbidity were monitored. This study detected no weight loss or apparent toxicity in polysaccharide-treated mice along the whole *in vivo* assays. Polysaccharide treatment lasted for 20 days, and the euthanasia of the animals was performed 24 h after the last drug administration. Then, tumor xenografts were extirpated, measured, and weighed, and a necropsy analysis involving tumors and distinct organs was carried out. Serum samples were obtained to quantify the circulating levels of creatinine, alanine aminotransaminase (ALT), and aspartate aminotransferase (AST) by colorimetric assay kits (Biosystems S.A., Barcelona, Spain).

### Fungal strain

The *G. parvulum* strain used in this study is a wild isolate previously collected in Puerto Berrío, Antioquia, under Contract No. 235 for Access to Genetic Resources and Their Derivatives, signed by the Ministry of Environment and Sustainable Development of Colombia and the UdeA, and preserved in the culture collection of the Biopolimer research group. The specimen was deposited in the herbarium under code HUA-191249 and preserved in laboratory conditions on potato dextrose agar (PDA) plates and subcultured every two months.

### Production and extraction of polysaccharides

The production and extraction of *G. parvulum* polysaccharides were performed according to the methodology proposed by López-Legarda et al. [15–17]. Briefly, the fungal strain was subcultured in agar plates with the ligninolytic Kirk media for one week. Then, it was incubated in five flasks of 100 ml of volume (pre-inoculum) and subsequently scaled up in a BIO-STAT® A plus bioreactor of 5 l by batch submerged cultivation using a ligninolytic patented medium. The fermentation conditions were 30 ± 1 ◦C, pH 4.5, 300 rpm, and 1.5 vvm for three days. At the end of the fermentation, the fungal mycelia were separated from the liquid phase using filtration followed by centrifugation. Mycelia were alcohol-defatted and broken using hot water extraction (HWE) followed by ultrasound-assisted extraction (UAE) to extract the IPS water-soluble fraction. The suspension was centrifuged, and the supernatant was concentrated and precipitated upon adding 4 vol of cold anhydrous ethanol and stored at 4°C overnight. The resulting precipitate was collected, washed, and centrifuged again. Thereafter, it was lyophilized to yield the crude IPS. On the other hand, to extract EPS water-soluble fraction, the filtrated culture broth was concentrated under reduced pressure, and the extraction process continued as described above to obtain the IPS. As a result, two water-soluble fractions, GIPS (*Ganoderma* intra-polysaccharide) and GEPS (*Ganoderma* exopolysaccharide), were obtained.

### Composition of crude polysaccharide extracts (GEPS and GIPS)

Characterization of the fungal polysaccharides was performed following protocols previously developed by López-Legarda et al. [15–17]. Total polysaccharide content of GIPS and GEPS was determined using a modified sulfuric acid method with glucose as a standard. β-Glucan content in each fraction was quantified using a mushroom- and yeast-specific β-glucan assay kit (Megazyme International, Wicklow, Ireland). Protein content was measured by the Bradford assay (Bio-Rad). All absorbance readings were taken on a UV–VIS spectrophotometer (Varian Cary 50 Bio), and each analysis was performed in triplicate.

### Cell culture and cell lines

MDA-MB-231 (estrogen-receptor-negative) human breast cancer cells, A549 human lung cancer cells, U937 human leukemic cells, EL4 mouse lymphoma cells, LLC mouse Lewis lung carcinoma, and RAW 264.7 mouse macrophage cells were obtained from the American Type Culture Collection (ATCC, Manassas, VA, U.S.A.). Adherent cells were grown in DMEM medium, and suspension cells were grown in RPMI-1640 medium containing 10% (v/v) fetal bovine serum (FBS), 100 units/ml penicillin, and 100 μg/ml streptomycin and maintained in an incubator at 37°C in a humidified atmosphere with 5% CO_2_. Cells for experiments displayed >95% viability.

### Cell growth inhibition assay

Cell proliferation and viability were assessed using the MTT (3-(4, 5-dimethylthiazolyl-2)–2, 5-diphenyl tetrazolium bromide) assay, which is based on the conversion of MTT into formazan crystals by living cells, which determines mitochondrial activity. Briefly, 3 × 10^3^ cells in 100 μl in 96-well flat-bottomed microliter plates were incubated in a complete medium and left to adhere for 12 h. For suspension cells, cells were allowed to grow for 4 h before experiments. Next, the culture medium was removed, and 100 µl of different concentrations of GIPS and GEPS extracts (12.5, 50, 100, and 200 µg/ml) or only medium (untreated control) was added to each well. After 72 h incubation, MTT (2 mg/ml, 10 μl) was added to each well and incubated for 4 h, as we previously described [15,16]. The unreduced MTT solution was discarded, and DMSO (100 μl) was added to each well to dissolve the purple formazan crystals. Measurements were done in independent triplicates. Absorbance was measured using a spectrophotometric microplate reader (Bio-Rad Laboratories Inc., U.S.A.) at 570 nm. The fraction of viable cells was calculated relative to the control cells based on the following equation: growth inhibition rate = (Absorbance sample*/*Absorbance control) × 100%. Each determination was performed in triplicate.

### Analysis of apoptosis-like cell death by flow cytometry

A total of 2 × 10^5^ cells were incubated in the absence or presence of 100 µg/ml of polysaccharides for 48 h and then analyzed for DNA breakdown by flow cytometry, staining with 50 μg/ml RNAse and 5 μg/ml propidium iodide in PBS, using a fluorescence-activated cell sorting (FACS) Calibur flow cytometer (Becton Dickinson, San Jose, CA), as previously described [18,19]. Quantitation of apoptotic-like cells was monitored following cell cycle analysis as the percentage of cells in the sub-G0/G1 region representing hypodiploid or apoptotic-like cells. Apoptotic cells are distinguished from non-apoptotic cells by measuring the percentage of cells in the sub-G0/G1 region of the cell cycle. In cell cycle analysis, the quantitation of apoptotic cells was calculated as the percentage of cells in the sub-G1 region (hypodiploidy).

### Preparation of conditionate medium of RAW264.7 cells treated with polysaccharides

RAW 264.7 macrophages were incubated in a DMEM medium with 25 and 50 µg/ml of each polysaccharide fraction for 24 h. The co-cultured medium was collected, filtered through a Millipore membrane filter with an average pore diameter of 0,22 µm, and stored at −20°C for future research [20]. Following the treatment of lung cancer cell lines with conditioned medium in different proportions, MTT was used to assess the growth of the cells. The fraction of viable cells was calculated relative to the control cells based on the following equation: growth inhibition rate = (Absorbance sample*/*Absorbance control) × 100%. Each determination was performed in triplicate.

### Nitric oxide quantification

Nitric oxide (NO) secretion was measured using the Griess reagent as previously described [21]. A total of 3 × 10^3^ cells were seeded in 96-well plates and left to adhere for 24 h. After cells were stimulated with 10 µg/ml LPS, 1 h later, polysaccharides (25 µg/ml) were added and cultivated for 24 h. Subsequently, the nitrite concentration in the culture medium was quantified using the Griess reagent by mixing equal volumes of the treated samples and the medium. Finally, after 15 min, the absorbance was read at 540 nm. LPS-stimulated or non-treated cells were used as a control.

### Immunomodulatory properties of polysaccharides of *G. parvulum*

The immunomodulatory capacity of polysaccharides of *G. parvulum* in RAW 264.7 macrophages was determined by culturing 3 × 10^5^ cells in six-well plates in a total volume of 2 ml culture medium. Cells were treated with 10 µg/ml LPS for 1 h to stimulate inflammatory responses. After 1 h of incubation, polysaccharides were added. After 24 h, the CBA mouse inflammation kit determined the concentration of TNF-α, IFN-γ, IL-6, IL-10, and MCP-1 in the supernatant through flow cytometry. Non-treated or LPS-stimulated cells were used as controls.

### RNA extraction and quantitative real-time PCR

mRNA levels in RAW 264.7 macrophages treated with polysaccharides of *G. parvulum* were determined by real-time PCR, following previous protocols [22,23].

RNA extraction was performed using TRIZOL reagent (ZYMO RESEARCH) to lyse the cells and obtain RNA. Following the manufacturer’s instructions, RNA purification was carried out using Zymo-Spin™ columns, with prewash and wash buffer washes, followed by sample resuspension in 20 µ DNase-free water. Subsequently, the RNA was quantified by spectrophotometry, and RNA integrity was ensured by the RNA Integrity Number (RIN) >8 with the Agilent 2100 Bioanalyzer (Agilent Technologies, Germany). RNA samples were treated with DNase to eliminate genomic DNA contamination. For qRT-PCR, cDNA was synthesized from RNA isolated from RAW 264.7 macrophages treated with PFs using the iScript cDNA synthesis Kit (Bio-Rad). Two microliters of isolated RNA were used for cDNA synthesis. The sample was then transferred to the thermocycler with a final volume of 20 µl under the following conditions: preheating at 25°C for 5 min, reverse transcription at 46°C for 20 min, RT inactivation at 95°C for 1 min, and cooling at 4°C for the required time. The primer sequences used to detect mRNA are listed in Table 1. The relative gene expression levels obtained from reverse transcription qRT-PCR were calculated using the ΔΔCt method after normalization to beta-actin (β-actin).

**Table 1:** Primers used for real-time quantitative PCR

Gen	Forward primers 5′-3 ′	Reverse primers 3 ′-5′
IL-1β	TCGCTCAGGGTCACAAGAAA	CATCAGAGGCAAGGAGGAAAAC
IL-6	TCTATACCACTTCACAAGTCGGA	GAATTGCCATTGCACAACTCTTT
TNF-α	CCCTCACACTCAGATCATCTTCT	GCTACGACGTGGGCTACAG
iNOS	GTTCTCAGCCCAACAATACAAGA	GTGGACGGGTCGATGTCAC
ARG1	CTCCAAGCCAAAGTCCTTAGAG	GGAGCTGTCATTAGGGACATCA
Fizzy1	CCAATCCAGCTAACTATCCCTCC	ACCCAGTAGCAGTCATCCCA
Mrc1	AGGGACCTGGATGGATGACA	TGTACCGCACCCTCCATCTA
β-actin	GGCTGTATTCCCCTCCATCG	CCAGTTGGTAACAATGCCATGT

### Establishment of the M1 macrophage and tumor-associated macrophages (TAMs)-like model

LLC cells were cultured in DMEM supplemented with 10% FBS until reaching 90% confluence. The medium was then changed to FBS-free, and the cells were cultured for 24 h. The supernatant was collected, labeled as tumor-conditioned medium (CoMe-LLC), and stored at −80°C until use. For polarization, RAW264.7 macrophages were stimulated with LPS (10 µg/ml) to induce classical activation (M1 polarization) or with IL-4 (20 ng/ml) plus CoMe-LLC (1 ml) to induce TAMs or alternative polarization (M2 polarization) for 24 h. Cells of both phenotypes were examined using quantitative RT-PCR (qRT-PCR) to measure the expression of mRNA transcripts related to the phenotype.

### Statistics

All experiments *in vitro* were repeated in triplicate with 3 independent replicates, and the results were expressed as the mean ± standard deviation (SD). Statistical analysis was conducted in GraphPad Prism Version 6 (GraphPad Software Inc., U.S.A.). The normal distribution of data was evaluated using the Shapiro–Wilk test. Data groups were compared using a two-tailed Student’s *t*-test. Differences between groups were considered statistically significant if *P*<0.05. The statistical significance is denoted by asterisks (*P<0.05; **P<0.01; ***P<0.005).

## Results

### Composition of crude polysaccharide extracts (GEPS and GIPS)

Table 2GEPS and GIPS from *Ganoderma parvulum* exhibit the high carbohydrate content characteristic of *Ganoderma*-derived polysaccharide preparations (99 ± 6% for GEPS and 88 ± 5% for GIPS). Notably, the total glucan content is significantly higher in GEPS (39.63 ± 1.3%) compared with GIPS (9.65 ± 0.5%), a difference largely attributable to the β-glucan fraction (33.98 ± 1.3% in GEPS vs. 7.99 ± 0.2% in GIPS). This predominance of β-glucans is consistent with previous reports indicating that β-D-glucans are the major bioactive components in *Ganoderma* extracts [24]. The α-glucan content remains low in both extracts (5.66 ± 0.4% in GEPS and 1.67 ± 0.4% in GIPS), and protein contamination is negligible—undetectable in GEPS and only 1 ± 0.003% in GIPS—indicating minimal presence of non-polysaccharide macromolecules. Given that both GEPS and GIPS consist almost entirely of carbohydrates with minimal impurities, their biological activity is likely attributable to their polysaccharide components. Overall, these findings are in good agreement with prior studies on *Ganoderma* polysaccharides, confirming that our extraction and purification method yields a β-glucan–rich product comparable with those previously reported [17].

### Inhibition of EL-4 tumor growth by polysaccharides of *G. parvulum*

**Figure 1:**
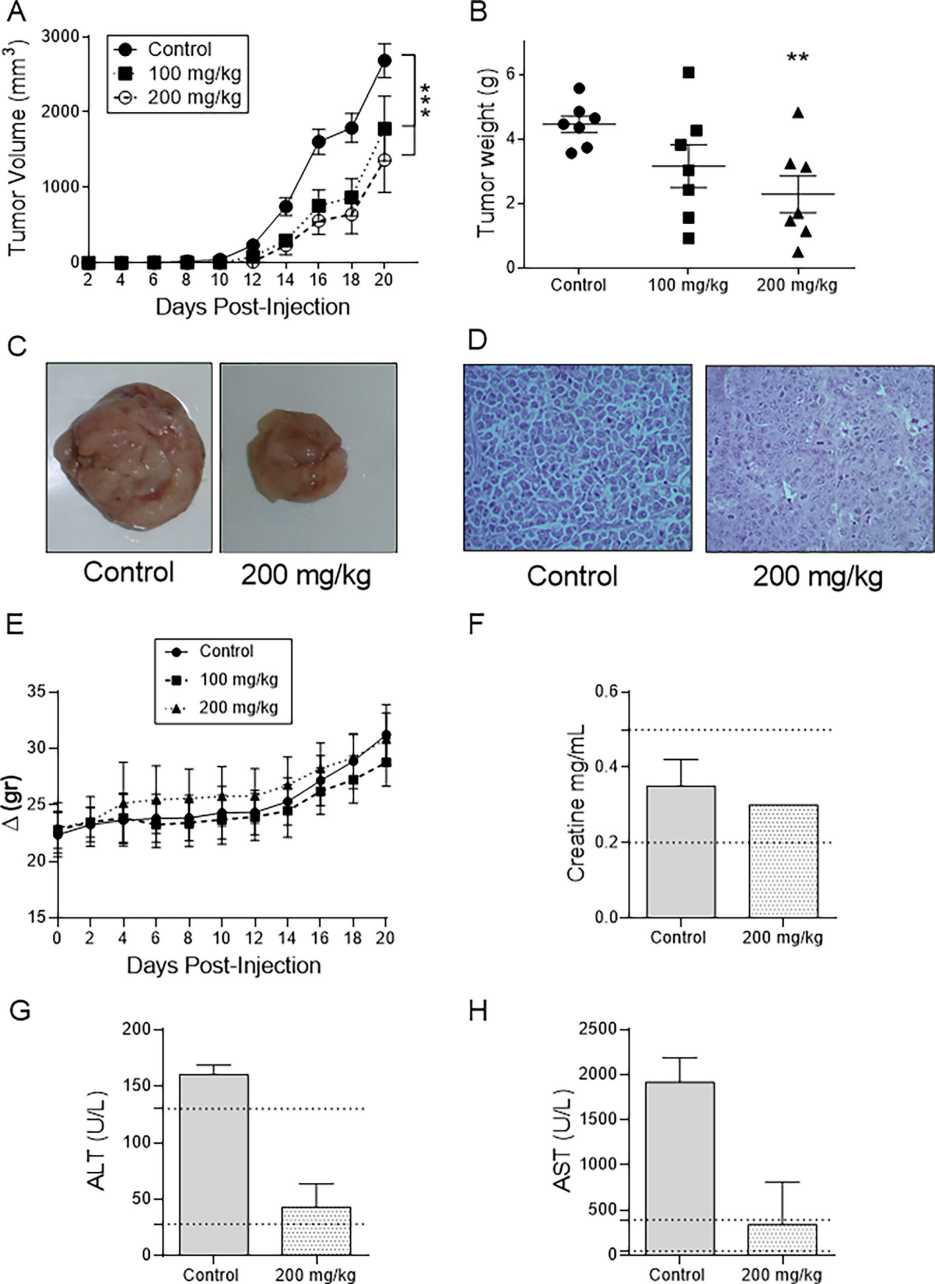
Inhibition of EL-4 tumor growth by polysaccharides of *G. parvulum*. Six-to eight-week-old C57BL/6 female wild-type (*N* = 7) were implanted with EL-4 cells, when tumors were palpable, mice were randomly assigned into each group and then treated with polysaccharides of *G. parvulum* (100 and 200 μg/kg/day) or PBS intragastrically for 20 days. At the end of the experiment, the tumor was removed and weighed. (**A**) Tumor volume of mice as a function of time. (**B**) Tumor weight of each group, (**C**) photographs of the tumor at the end of the experiment,(**D**) pathological observation with H&E staining of EL-4 tumor sections, (**E**) body weights of the mice over the entire experimental period from the day of tumor injection. The serum biochemical examination in mice control and 200 mg/kg/day were evaluated at the end of the experiment; (**F**) creatine levels, (**G**) ALT levels, and (**H**) AST levels. *P<0.05, **P<0.01, ***P<0.005, and ****P<0.001 significantly different from control.

According to previous studies, polysaccharides from *Ganoderma spp*. can inhibit the growth of mice tumors [25]. We used crude polysaccharides from *G. parvulum* to evaluate the anti-tumor activity on mice EL-4 (lymphoma) tumor models by oral administration. Polysaccharides had a statistically significant effect on EL-4 volume tumor (Figure 1A). As seen in Figure 1B, polysaccharides decreased the EL-4 tumor weight to a noteworthy level of 100 µg/ml, with an inhibition ratio of 30%. While a 200 µg/ml dose significantly reduces tumor weight, with an inhibition ratio of 50% (**P<0.005).

We collected tumors from control and 200 mg/kg groups for H&E staining and visual observation to investigate morphology and pathology in polysaccharide-treated tumors (Figure 1C–D). In the control groups’ tumor tissue, nucleated cells with large nuclei and dense chromatin were observed, suggesting rapid tumor growth. At the same time, polysaccharides of *G. parvulum* treatment decrease the number of tumor cells with dense chromatin.

Polysaccharide-treated mice (100 and 200 mg/kg/day) did not show any clinical signs of toxicity immediately or during treatment, indicating that polysaccharides were non-toxic. Figure 1E shows the changes in body weight during the administration. In both dose groups, values were comparable with those of the control group, and no significant differences were noted. Consistent with earlier studies, serum biochemistry revealed normal creatinine levels across all groups, while elevated ALT and AST levels were observed only in the control group (Figure 1F–H) [26].

### Polysaccharides of *G. parvulum* decrease cancer cell viability without inducing cell death

**Figure 2:**
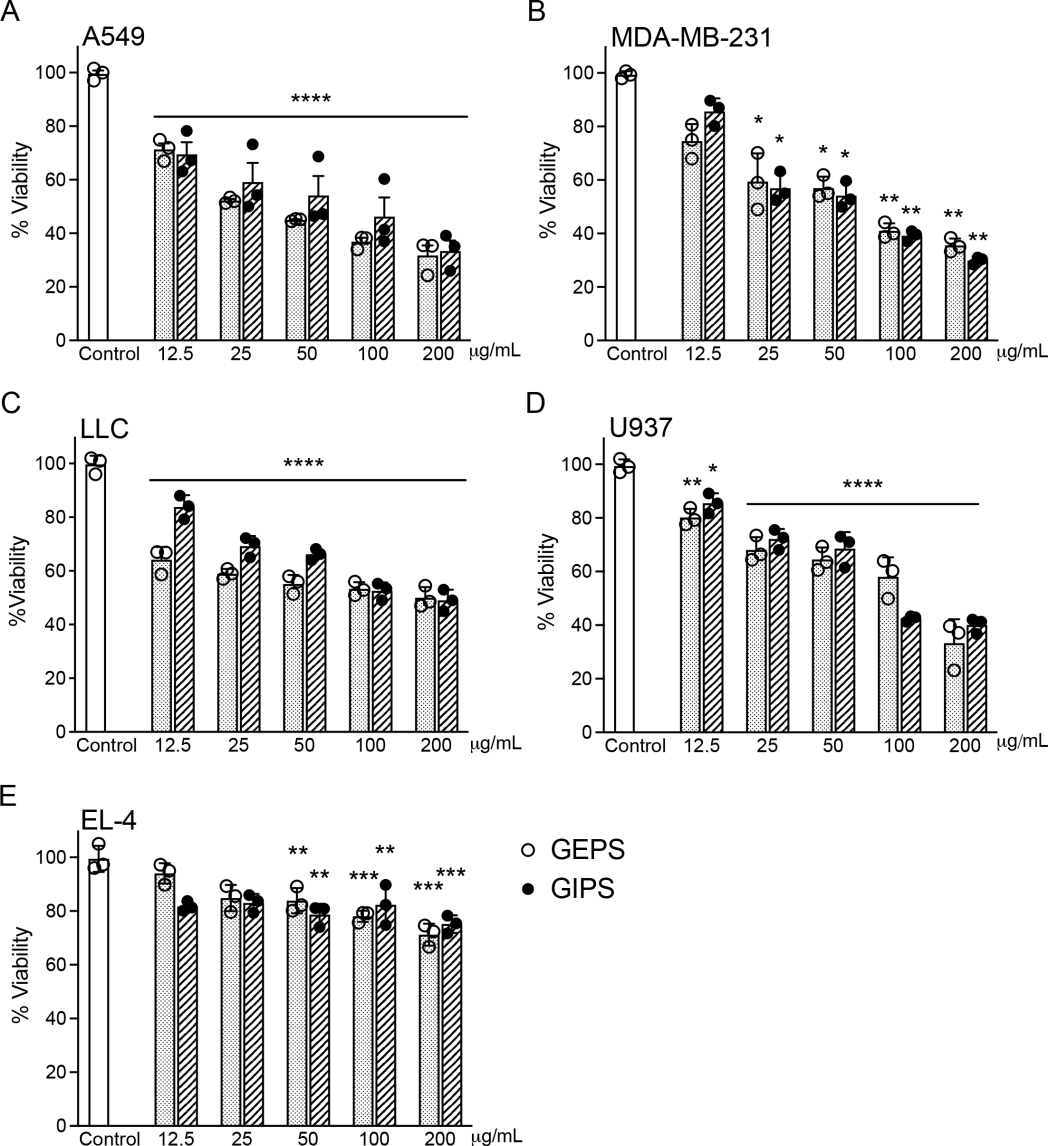
Effect of *G. parvulum* soluble polysaccharides (GEPS and GIPS) on the proliferation of tumor cell lines. The proliferation rate of the (**A**) A549, (**B**) MB-MBA-231, (**C**) LLC, (**D**) U937, and (**E**) EL-4 cell lines. Cells were incubated for 72 h. Control cells were incubated with medium alone (untreated cells). The cell viability was evaluated using an MTT assay. Data were expressed as mean ± SD (*n* = 3). *P<0.05, **P<0.01, ***P<0.005, and ****P<0.001 significantly different from control.

To assess the effectiveness of GPs in killing cancer cells, colorimetric MTT assays were conducted on A549 human lung cancer cells, MDA-MB-231 (estrogen-receptor-negative) human breast cancer cells, U937 human leukemic cells, LLC mouse Lewis lung carcinoma cells, and EL4 mouse lymphoma cells. In A549, MDA-MB-231, U937, and LLC cells, as well as to a lesser extent in EL-4 cells, there was a dose-dependent effect on cell viability (Figure 2A–E). Polysaccharide treatment resulted in consistently low cell viability percentages across all tested concentrations on MDA-MB-231 (50–100 μg/ml), A549 (25–50 μg/ml), U937 (100–200 µg/ml), and EL-4 cells ( > 200 µg/ml). There is no statistically significant difference between treatment with GIPS and GEPS. However, GEPS was more toxic than GIPS for lung cancer cells, for A549 cells at 25 µg/ml (*P*<0.01), 50 µg/ml, and 100 µg/ml (*P*<0.001) concentrations, while for LLC cells at 12 µg/ml, 25 µg/ml, and 50 µg/ml (*P*<0.0001) concentrations. Cell viability of EL-4 cells did not show a marked decrease with increasing concentrations of GIPS and GEPS (Figure 2E). However, it is noteworthy that cell viability remained below the 80% threshold typically considered as the limit for cytotoxicity. No significant changes were observed in experiments performed on normal cells, such as VERO cell lines and peripheral blood mononuclear cells (PBMCs) (Supplementary Figure S1). Polysaccharides were subsequently tested to see if they could induce the death of A549 and MDA-MB-231 cells with apoptotic characteristics. In that regard, the amount of DNA was measured by flow cytometry. A549 and MDA-MB-231 cells treated with 100 µg/ml of GEPS, or GIPS, showed poor apoptosis cell death after 24 h of incubation (Supplementary Figure S2).

### Polysaccharides of *G. parvulum* stimulate the secretion of pro-inflammatory cytokines in the macrophage

**Figure 3:**
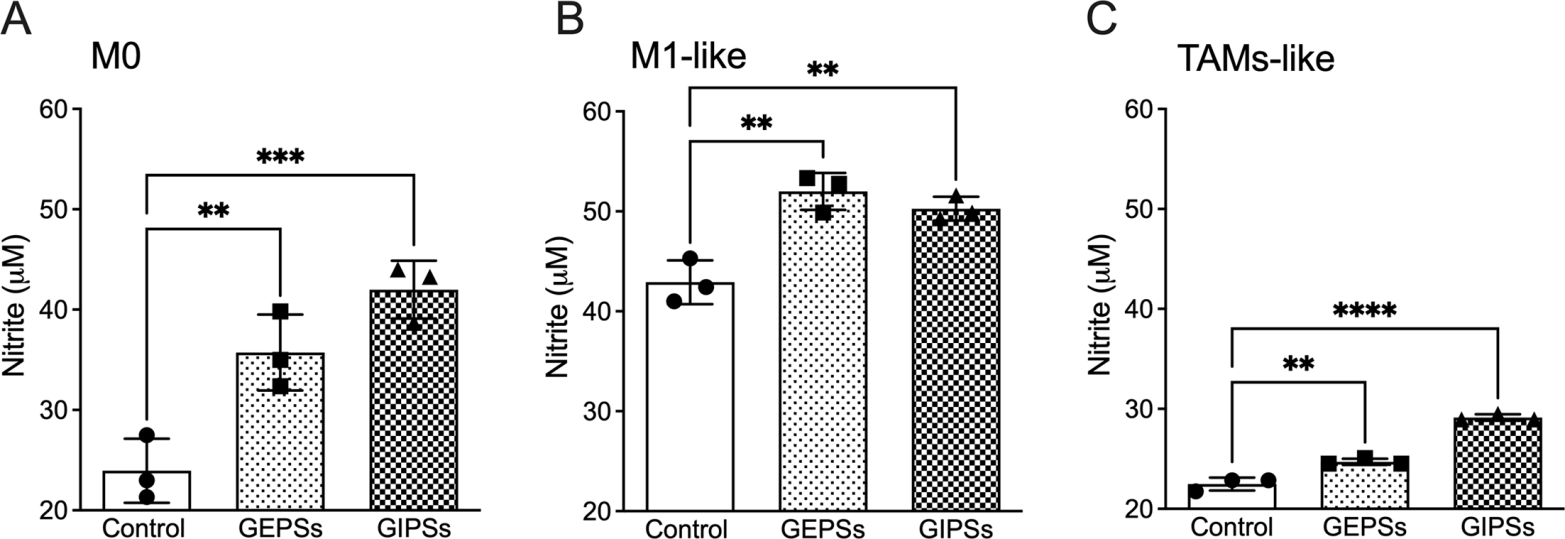
Effect of soluble polysaccharides of *G. parvulum* on nitric oxide production in RAW264.7 polarized macrophages. (**A**) M0 macrophages, (**B**) M1 macrophages pre-treated with LPS, (**C**) TAMs-like M2 macrophages pre-treated with IL4+CoMe LLC for 24 h later with 25 µg/ml of polysaccharides until they were 24 h older. Nitrite production was assessed using the griess reagent. Data were expressed as mean SD (*n* = 3). * *P*<0.05, **P<0.01, ***P<0.005, and ***P<0.001 significantly different from the control.

**Figure 4:**
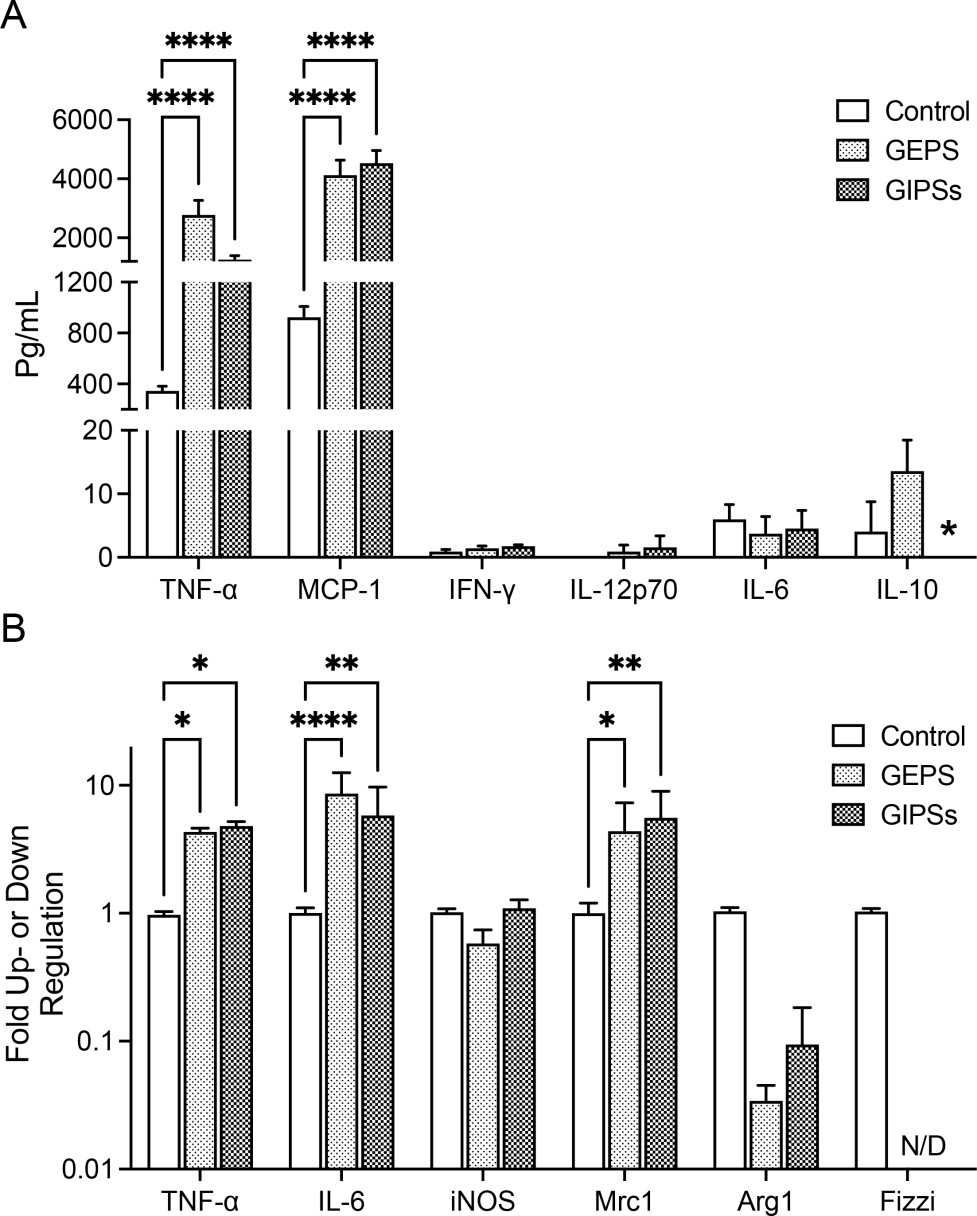
Effect of soluble polysaccharides from *G. parvulum* (GEPS and GIPS) on the repolarization in RAW264.7 macrophages. (**A**) Production of pro-inflammatory cytokines and (**B**) measurement of genes activation proteins in M0 macrophages. The supernatant was used to make the measurement by CBA. Total RNA was obtained for PCR in real-time assay. Data were expressed as mean SD (*n* = 3). *P<0.05, **P<0.01, ***P<0.005, and ****P<0.001 significantly different from the control.

The immunomodulatory activity of GEPS and GIPS samples was analyzed to determine their ability to influence the secretion of NO and cytokines from macrophages. In this sense, we investigated the effect of GIPS and GEPS on the macrophages by quantifying the secretion of NO and cytokines after 24 h of treatment with 25 µg/ml. Both GEPSs and GIPS increased the levels of NO on M0 (GEPS *P*<0.05 and GIPS *P*<0.005), M1 (GEPS *P*<0.05 and GIPS *P*<0.05), and TAMs-like (GEPS *P*<0.05 and GIPS *P*<0.001) macrophages (Figure 3A). Besides, GEPS increases the levels of TNF-α (*P*<0.0001) and MCP-1 (*P*<0.0001). While GIPSs increase the levels of TNF-α (*P*<0.0005) and MCP-1 (*P*<0.0001) and decrease IL-10 (*P*<0.05) (Figure 4A). Both polysaccharide treatments did not affect the immunomodulatory cytokine IL-12p70, IL-6, and IFN-γ. Subsequently, we validated TNF-α gene expression (Figure 4B). Additionally, we found that both GEPS and GIPS induced the expression of IL-6 (*P*<0.0005) and Mrc1 (*P*<0.05) while reducing the induction of Arg1 (*P*<0.0001) and Fizzi (at undetectable levels) (Figure 4B). We know that M2 macrophages are characterized by the expression of Arg1, a crucial enzyme in the urea cycle, as it catalyzes the hydrolysis of arginine to produce ornithine. Ornithine, in turn, acts as a substrate for ornithine decarboxylase (ODC). This cellular pathway regulates fundamental aspects such as DNA replication, protein translation, cell growth, and differentiation [27]. In this study, we observed that all fungal polysaccharides (FPs) reduced the expression of Arg1. This finding suggests that the FPs can activate macrophages through the classical pathway, allowing them to perform pro-inflammatory functions. It suggests that polysaccharides enhanced the activity of macrophages by activating them toward an M1 polarization.

### Conditionate medium of RAW 264.7 cells treated with polysaccharides of *G. parvulum* decreases the viability of cancer cells

**Figure 5:**
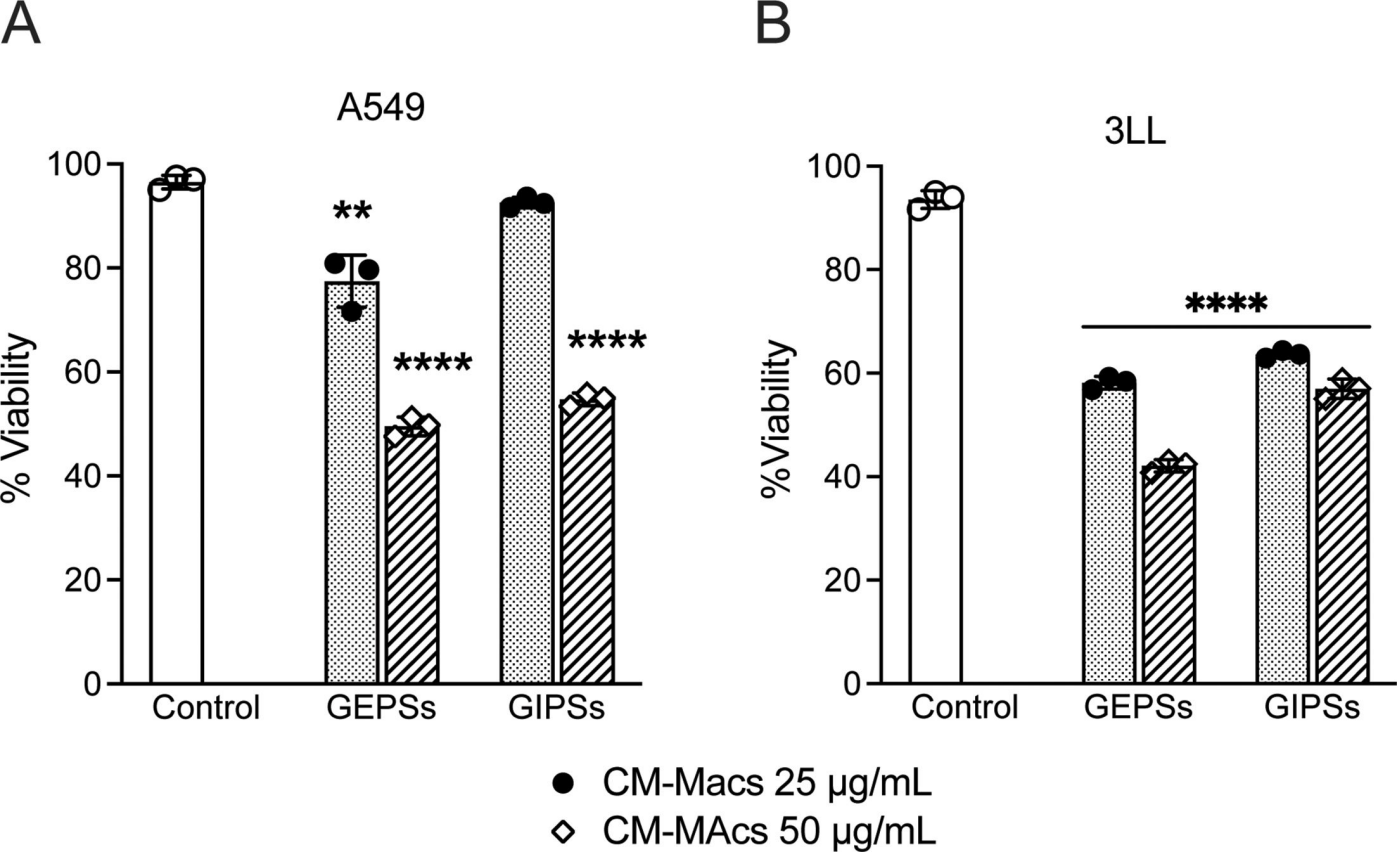
Effect of CoMe-MGp-Ps on tumor line viability. The cells were treated with two different proportions of the conditioned medium at two proportions, 50:50 or 100%. Cells were incubated for 72 h. Control cells were incubated with medium only. Cell viability was evaluated by the MTT test. Data were expressed as mean SD (*n* = 3). *P<0.05, **P<0.01, ***P<0.005, and ***P<0.001 significantly different from the control.

Macrophage-conditioned medium (CM) has been known to reflect macrophage functions [28–30]. For example, it has been shown that CM directly promoted fibroblast proliferation and the production of extracellular matrix proteins *in vitro [29]*. Therefore, to understand the functional implications of its activation by polysaccharides, we evaluated the effect of the conditioned medium of macrophages treated with polysaccharides of *G. parvulum* (CoMe-MGp) on the viability of tumor cells. To this end, an MTT colorimetric assay was carried out in human lung cancer cells A549 and mouse Lewis lung carcinoma LLC stimulated with CoMe-MGp at concentrations of 25 and 50 µg/ml. The cells were treated at a 50:50 ratio (DMEM+10% FBS: CoMe-MGp) with conditioned media of macrophages treated at both concentrations; conditioned medium from untreated cells was used as the control. A dose-dependent effect on cell viability was observed (Figure 5A–B). When comparing concentrations (25 and 50 µg/ml), greater activity was observed at concentrations of 50 µg/ml, especially highlighting GEPS (*P*<0.0001) in both cell lines. These results suggest that the cytokines exhibited by macrophages, when treated with PFs, present anti-tumor activity. By extrapolating this theory from what is documented in the literature, it could be argued that this function is linked to TNF-α in the supernatant [31].

### Polysaccharides of *G. parvulum* modulated expression of inflammasome-related genes

**Figure 6:**
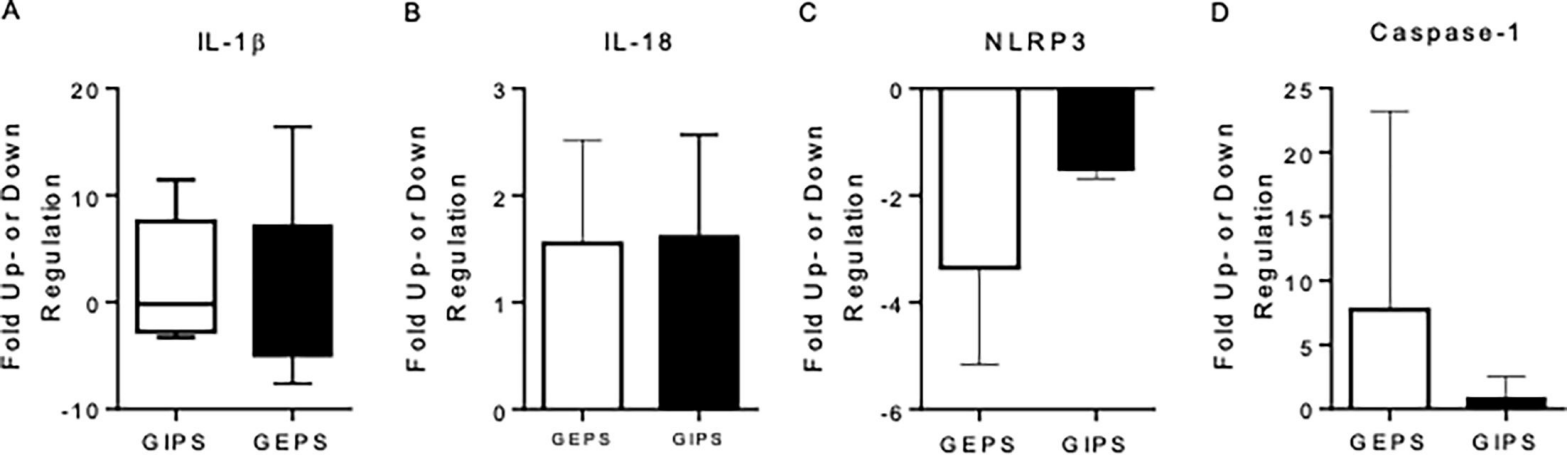
mRNA expression of inflammasome-related genes RAW 264.7 macrophages treated with soluble polysaccharides of *G. parvulum* . RNA samples obtained from macrophages with polysaccharides of *G. parvulum* were quantified by real-time PCR: (**A**) IL-1 *β,* (**B**) IL-18, (**C**) NLRP3, and (**D**) caspase-1. GAPDH was used as the constitutive gene to normalize the RNA content. Results are representative of at least three independent experiments by triplicate.

To investigate the effect of polysaccharides in regulated inflammasome-related genes, RAW 264.7 macrophages were treated for 6 h with GEPS or GIPS (25 µg/ml). Here, we found that IL-1β, IL-18, and Caspase-1 mRNA levels were positively regulated, and NLRP3 was negatively regulated in macrophages treated with both GEPS and GIPS compared with control cells (Figure 6A–C).

### Polysaccharides of *G. parvulum* enhanced the secretion of pro-inflammatory cytokines in LPS-induced RAW 264.7 macrophages

**Figure 7:**
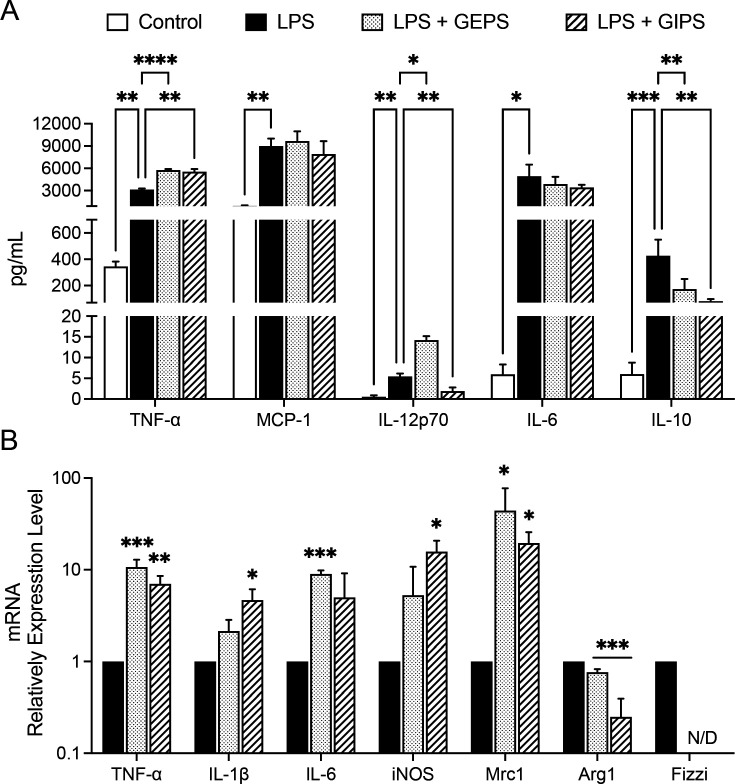
Effect of soluble polysaccharides from *G. parvulum* (GEPS and GIPS) on the repolarization in RAW264.7 macrophages. (**A**) Production of pro-inflammatory cytokines in supernatant by CBA assay(B) Effect of the polysaccharides in gene expression of activation proteins in M1 macrophages using PCR in real-time. Results are representative of at least three independent experiments by triplicate Data were expressed as mean SD (*n* = 3). *P<0.05, **P<0.01, ***P<0.005, and ***P<0.001 significantly different from the control.

LPS is a known inducer of inflammatory cytokines in immune cells. Once macrophages are activated in the presence of LPS, a naturally high pro-inflammatory content such as NO and cytokines is produced. The effect of polysaccharides was investigated in macrophages primed with LPS (10 µg/ml), then treated for 24 h with either GEPS or GIPS at 25 µg/ml. As seen in Figure 3B, NO production was significantly enhanced by GEPS and GIPS (*P*<0.005). Compared with the non-stimulated cells, stimulation with LPS generates the secretion of the cytokines, such as IL-12p70, TNF-α, INF-γ, MCP-1, IL-10, and IL-6 in RAW264.7 macrophages (Figure 7A). The results showed that treatment with GEPS was able to enhance TNF-α (*P*<0.005) and IL-12p70 (*P*<0.01) and significantly reduce IL-10 (*P*<0.01) in LPS-primed cells. In addition, GIPSs significantly enhanced TNF-α (*P*<0.01) and IL-12p70 (*P*<0.01) and reduced IL-10 (*P*<0.005) and IL-6 (*P*<0.05) (Figure 7A). Additionally, when observing how the FPs modulated macrophages (Figure 7B), it was found that GEPS induced the production of IL-6 (*P*<0.005), GIPS induced the production of IL-1β (*P*<0.05), Mrc1 (*P*<0.05), and iNOS (*P*<0.05) (Figure 7B). Both polysaccharides induced the production of TNF-α (*P*<0.005 and *P*<0.01, respectively) and reduced the induction of Arg1 (*P*<0.005) (Figure 7B). The expression of Fizz1 was not detected (Figure 7B). This finding demonstrates that FPs can activate M1 macrophages through the classical pathway, allowing them to perform anti-tumor functions.

### *G. parvulum* polysaccharides promoted the repolarization of TAMs-like in M1 macrophages

**Figure 8:**
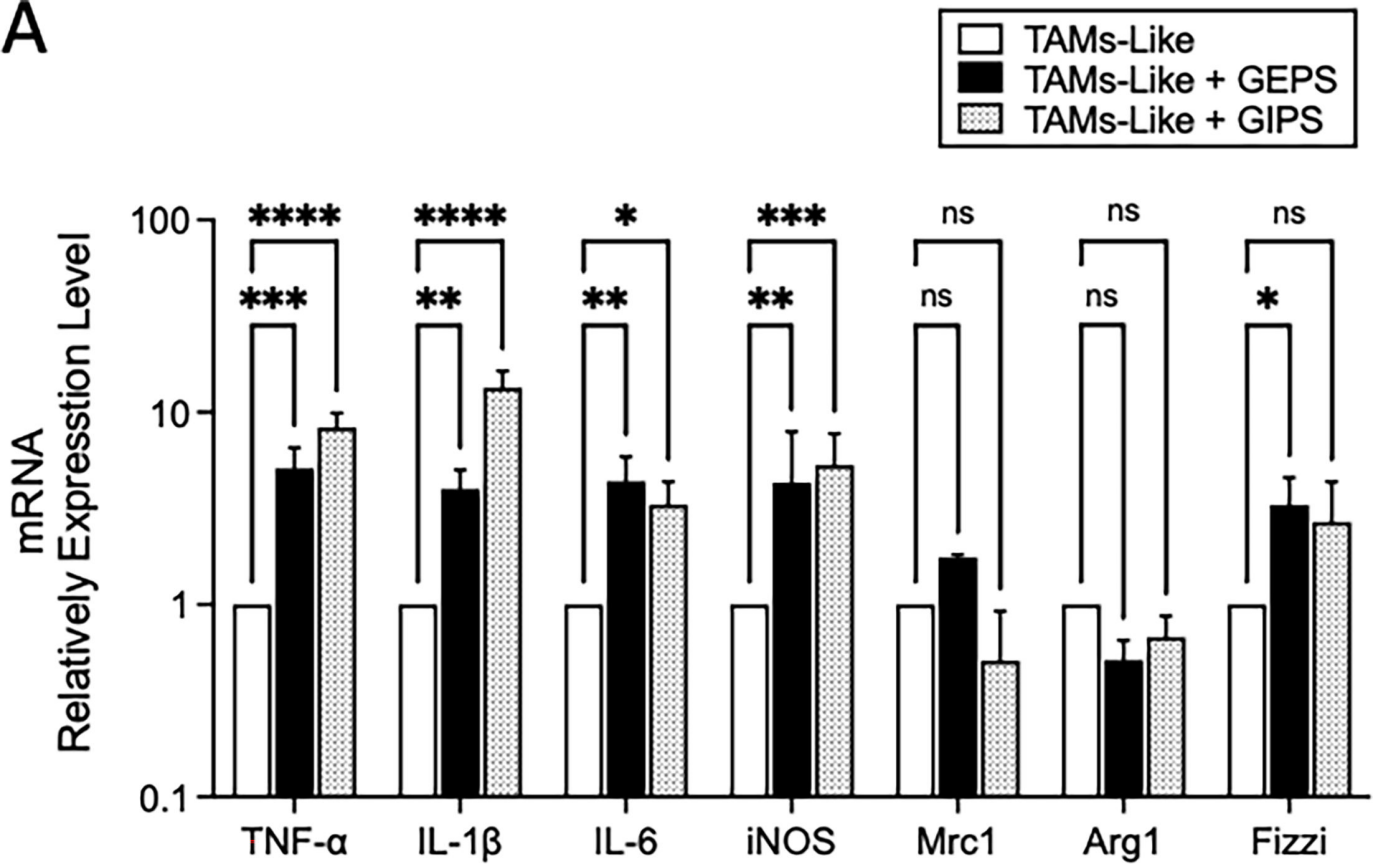
Effect of soluble polysaccharides from *G. parvulum *(GEPS and GIPS) on the repolarization in RAW264.7 macrophages. Macrophages are polarized to TAMs like M2. RNA samples obtained from macrophages treated with IL-4+CoMe LLC and subsequently with polysaccharides, the gene expression of activation genes were quantified by real-time PCR. β-actin was used as the constituent gene to normalize the RNA content. The results are tripled. Data were expressed as mean SD (*n* = 3). *P<0.05, **P<0.01, ***P<0.005, and ***P<0.001 significantly different from the control.

The immunomodulatory activity of FPs was analyzed in terms of their ability to influence the repolarization of TAMs-like (Supplementary Figure S3). These are characterized by being anti-inflammatory, tissue repair, precursors of resistance to treatment and tumor progression. In addition, the expression of cytokines such as IL-6, IL-10, TGFβ, and, to a lesser extent, TNF-α, and the expression of genes such as VEGF, Arg1, Fizzi1, among others [32]. In this sense, we investigated the effect of FPs on TAMs-like by quantifying the secretion of mRNA transcripts after 24 h of treatment with 25 µg/ml (Figure 8). *G. parvulum* polysaccharides, such as GEPS, induced the production of TNF-α (p-value 0.0004), IL-1β (*P*<0.00781), IL-6 (*P*=0.0027), and iNOS (*P*=0.0035) (Figure 8). While GIPS induced the production of TNF-α (*P*<0.0001), IL-1β (*P*<0.0001), IL-6 (*P*=0.042), and iNOS (*P*=0.0002) (Figure 8). In addition, MRC1 and Arg1 showed a downward trend after treatment with FPs (Figure 8). These results indicate that *G. parvulum* polysaccharides can repolarize macrophages to an M1 stage.

## Discussion

This study investigated the effect of crude polysaccharides extracted from *G. parvulum* on lymphoma growth. Our result revealed that these polysaccharides inhibited lymphoma growth and demonstrated no treatment-related clinical signs of toxicity. Furthermore, we obtained GEPS and GIPS by purifying these polysaccharides, inhibiting the proliferation of various tumor cell lines without inducing cell death.

To explore the immunoregulatory activity of GEPS and GIPS, we conducted experiments using RAW 264.7 macrophages. The results showed an increased production of nitric oxide, TNF-α, MCP-1, IL-10, and increased expression of IL-1β, IL-18, and Caspase-1, while NLRP3 was down-regulated. Additionally, GEPS and GIPS reduced the production of IL-10 and IL-6 in macrophages stimulated with LPS. These findings suggest that the anti-tumor effects of *G. parvulum* polysaccharides primarily stem from their ability to stimulate an immune response mediated by macrophages.

Recognizing that an imbalance between cell proliferation and apoptosis is a hallmark of tumors, targeting these features holds immense promise as a tumor treatment strategy. Immunotherapy has revolutionized cancer treatment [32], and polysaccharides from various sources, characterized by their molecular weight, solubility, glycosidic linkages, and administration routes, exhibit distinct mechanisms of action [10]. Our study aligns with this notion, and further investigations are imperative to unravel the intricate molecular mechanisms underlying polysaccharide-induced anti-tumor responses.

*Ganoderma* polysaccharides, with a history of use in traditional Chinese medicine, have proven their efficacy in tumor therapy with low toxicity [8,33–35]. They achieve this by suppressing tumorigenesis, inhibiting tumor growth, preventing metastasis, and modulating immune cells [33]. Although some studies suggest that fungal polysaccharides may not directly affect tumor cell lines at low concentrations, they play an indispensable role in activating the immune system and promoting immune cell maturation and differentiation. Consequently, while not recommended as first-line cancer treatment, Ganoderma polysaccharides have substantial potential as adjuvants to conventional therapies, enhancing tumor response and boosting host immunity [36,37].

This study aimed to evaluate crude polysaccharides from *G. parvulum in vivo* and water-soluble GIPS and GEPS *in vitro*. These polysaccharides were extracted from a strain isolated in the Colombian forest, previously identified, and characterized through classical and molecular taxonomy in our laboratory [17]. This choice was motivated by the low solubility of the extract. Our investigation into the *in vitro* effects of GIPS and GEPS on EL-4 cells revealed no inhibition of their proliferation, indicating a lack of direct harm. Conversely, crude polysaccharides from *G. parvulum* (administered at 100 and 200 mg/kg/day) exhibited significant inhibition of implanted lymphoma EL-4 cells. Previous studies suggest that *G. lucidum* polysaccharides can potentiate immunomodulatory activity *in vivo*, substantially contributing to their anti-tumor efficacy [34,38]. Importantly, the concentrations employed in our study proved non-toxic to the mice, as evident from the absence of alterations in mouse weight and serum biochemical parameters. These parameters indicate organ health, with elevated ALT and AST levels suggesting potential liver damage and creatinine levels reflecting kidney function [39,40]. Treatment with 200 mg/ml/day of polysaccharides reduced serum levels of creatinine, ALT, and AST compared with the control group, affirming the non-toxicity of *G. parvulum* polysaccharides when administered orally.

These findings further underscore the ability of GIPS and GEPS to inhibit the growth of various tumor cell lines. Cell proliferation was inhibited by over 50% in breast (MDA-MB-231), lung (A549), and leukemia (U937) cancer cells upon treatment with GEPS and GIPS extracts at lower concentrations (100 µg/ml). Notably, when assessing the effect of *G. parvulum* polysaccharides on mouse lymphoma cells *in vitro* (EL-4) after 72 h, none of the concentrations studied (ranging from 25 to 300 µg/ml) exhibited significant inhibitory effects. However, oral administration of polysaccharides (at 100 and 200 mg/kg/day) led to a significant reduction in tumor volume in an *in vivo* model.

Numerous studies have demonstrated that polysaccharides can manipulate the TME and induce anti-tumor responses by directly interacting with macrophages. Additionally, they may influence various other immune cell types, including macrophages, dendritic cells (DCs), natural killer cells (NK), and T and B lymphocytes [10]. Our study focused on evaluating the inflammatory response in macrophages *in vitro*, recognizing its significance within the TME. However, we acknowledge the need for future research to explore the broader impact of polysaccharides on diverse immune cell populations.

This study demonstrated that GEPS and GIPS effectively activate M1 macrophages, as indicated by increased production of NO, TNF-α, and MCP-1. TNF-α is recognized for its cytotoxic properties and plays a key role in the anti-tumor activity of macrophages [41].

Furthermore, we noted an increase in IL-10 levels induced by GEPS and a decrease in GIPS. Pro-inflammatory cytokines are naturally produced at high levels upon macrophage activation in the presence of LPS. We also examined how polysaccharides regulate cytokines when stimulated by LPS. To this end, macrophages were stimulated with LPS and subsequently treated with GEPS or GIPS. GEPS led to increased secretion of NO, TNF-α, and IL-12p70 cytokines in macrophages when co-treated with LPS, whereas GIPS increased the secretion of NO and TNF-α while reducing IL-12p70. Notably, both compounds diminished IL-10 production when macrophages were stimulated with LPS. This holds significance as IL-10 has a profound influence on promoting tumor development [42]. Considering these findings, it is apparent that *G. parvulum* polysaccharides can polarize macrophage responses, inhibiting tumor development.

The NLRP3 inflammasome, a collection of multimeric cytosolic proteins that assemble when cells are perturbed, plays a multifaceted role in tumorigenesis [43]. This activation, in turn, leads to the maturation and release of inflammatory cytokines, including interleukin (IL)-1β and IL-18, and contributes to inflammatory cell death [44]. Considering the role of inflammatory cytokines in inflammation-associated diseases’ pathogenesis [45], it’s evident that aberrant NLRP3 activation can modulate these conditions [46]. Tumorigenesis, encompassing processes like proliferation, invasion, angiogenesis, and metastasis, is intrinsically associated with inflammation. NLRP3’s impact on tumorigenesis is complex, demonstrating both anti-tumorigenic effects, notably observed in colitis-associated colorectal cancer (CAC), and tumor-promoting effects, as seen in gastric and skin cancers [47]. Recent research has highlighted alterations in NLRP3 inflammasome gene expression [47]. Additionally, human melanoma cells constitutively express and activate NLRP3, leading to autoinflammation through caspase-1 activity and the production of biologically active IL-1β [44]. These findings underscore the pivotal role of the NLRP3 inflammasome in cancer. In our study, we observed an up-regulation in the expression of mRNA levels for IL-1β, IL-18, and caspase-1 in RAW 267.4 macrophages treated with GEPS and GIPS. This suggests the potential of *G. parvulum* polysaccharides to activate the inflammasome. However, it is essential to note that further studies are warranted to explore this hypothesis comprehensively.

The balance between M1 and M2 macrophages holds paramount importance in cancer development. Monocytes infiltrate tumor tissues and differentiate under the influence of chemokines to become TAMs [48,49]. TAMs exhibit M2-like characteristics within the TME, promoting tumor growth. Only a limited subset of TAMs displays M1-like properties capable of suppressing tumor growth. Research has shown that M2-TAMs actively suppress immunity, fostering cancer cell growth, metastasis, and resistance to chemotherapy. Given these findings, targeting TAMs is a promising avenue for enhancing disease control across various cancer types. Consequently, numerous agents manipulating TAMs are either in clinical trials or poised for testing shortly.

**Figure 9:**
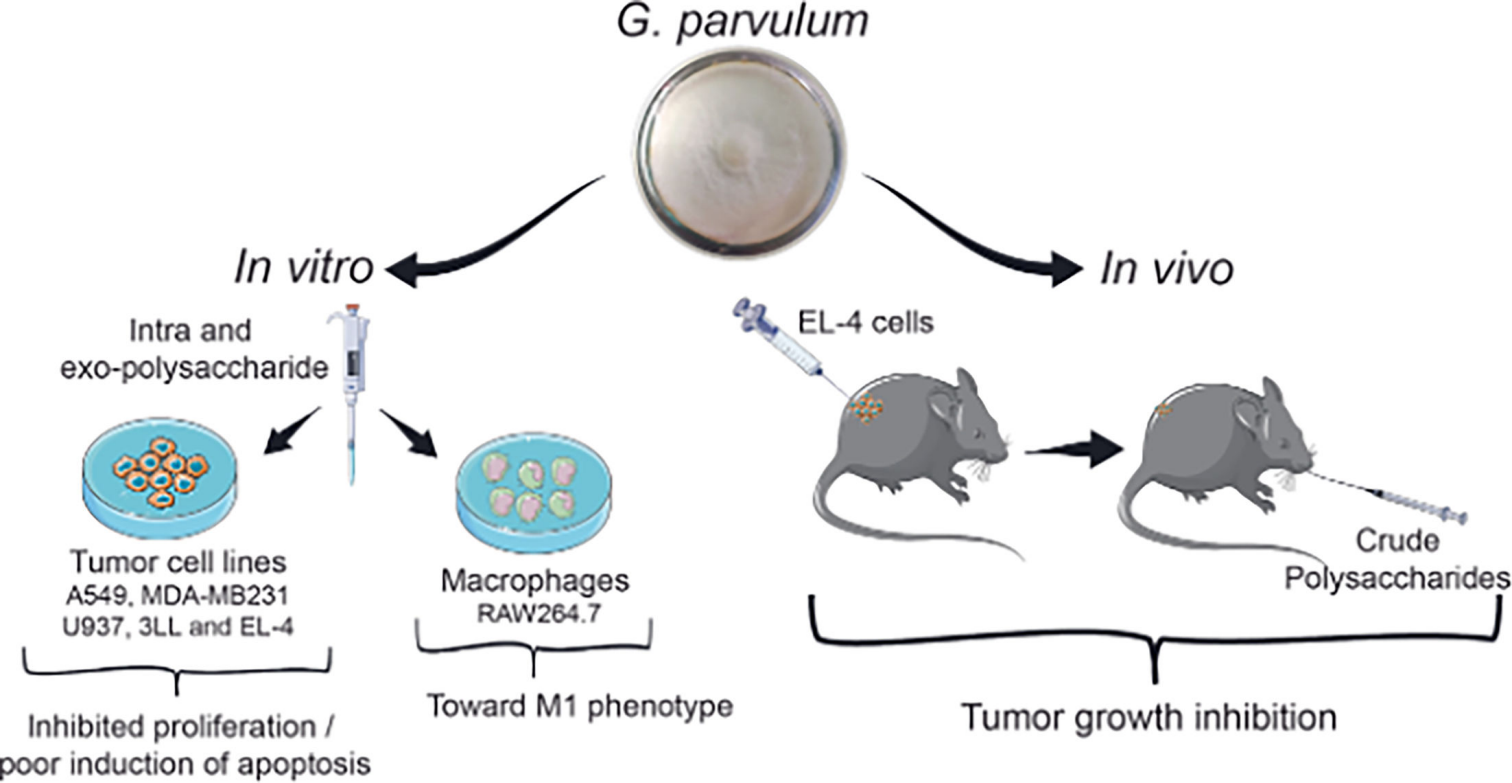
*G. parvulum* polysaccharides inhibit tumor growth *in vivo* without causing toxicity, suppress cancer cell proliferation, and activate macrophages. Polysaccharides may influence the function of tumor-associated macrophages (TAMs) within tumor microenvironments, thus suggesting their potential as immunomodulatory agents.

In conclusion, our study provides compelling evidence that *G. parvulum* polysaccharides inhibit tumor cell growth *in vivo* without inducing toxic effects. Moreover, they suppress cancer cell proliferation and can activate macrophages *in vitro* (Figure 9). We speculate that these polysaccharides might play a regulatory role in the balance between M1 and M2 TAMs within the TME *in vivo*. Therefore, further exploration into these immune cell populations in *vivo* cancer models is essential to fully elucidate their therapeutic potential in cancer treatment. Importantly, the *G. parvulum* strain used in this study is a wild isolate collected in Colombia, contributing to the growing body of knowledge on the country’s fungal biodiversity. The polysaccharides were biotechnologically produced under submerged cultivation, enabling enhanced yields, faster production times, controlled conditions, and scalability—key advantages for potential future industrial applications.

**Highlights**

- Immunotherapy is revolutionizing cancer treatment.
- Polysaccharides have gained considerable attention due to their anti-tumor and immunoregulatory properties.
- Polysaccharides may exert anti-tumor activity primarily through macrophage activation.

## Supplementary material

Online supplementary material 1

## Data Availability

The data presented in this study are available in the main text of the figures. Raw data are available on request from the corresponding author.

## Abbreviations

A549: Human lung carcinoma
ALT: Alanine aminotransferase
AST: Aspartate aminotransferase
DMEM: Dulbecco’s modified Eagle’s medium
DMSO: Dimethyl sulfoxide
DNA: Deoxyribonucleic acid
EL4: Lymphoma induced in a C57BL mouse
FBS: Fetal Bovine Serum
GEPS: *Ganoderma parvulum* exopolysaccharides
GIPS: *Ganoderma parvulum* intrapolysaccharides
HWE: Hot Water Extraction
IL-10: Interleukin 10
IL-18: Interleukin 18
IL-1β: Interleukin 1 beta
IPS: Intrapolysaccharides
LLC: Mouse Lewis Lung Carcinoma
LPS: Lipopolysaccharides
MCF-7: Human breast adenocarcinoma, estrogen receptor positive (ER⁺)
MCP-1: Monocyte Chemotactic Protein-1 (Human)
MDA-MB-231: Human breast adenocarcinoma, estrogen receptor negative (ER⁻)
NLRP3: NLR family pyrin domain containing 3
NO: Nitric oxide
PBS: Phosphate-buffered saline
PDA: Potato dextrose agar
RAW 264.7: Mouse macrophage cell line
RNA: Ribonucleic acid
ROS: Reactive oxygen species
RPMI: Roswell Park Memorial Institute
SIU: Sede de Investigación Universitaria
TNF-α: Tumor necrosis factor alpha
U937: Human histiocytic lymphoma
UAE: Ultrasound-Assisted Extraction
WT: Wildtype
