# Supplementary material for: Anti-tumor and immunomodulatory activity of Ganoderma parvulum-derived polysaccharides
Source: Biosci Rep. 2025 Oct 13;45(10):597–613. doi: 10.1042/BSR20240113 (PMC12784341; doi:10.1042/BSR20240113)
Supplement: Online supplementary material 1 [file bsr-45-10-BSR20240113-s001.docx]

Supplementary Figure 1

**Supplementary figure 1. Cell viability with MTT assay on normal cells.** (A) cell viability analysis of Vero cells and (B) macrophages RAW 264.7. The data were displayed as means ± S.D. of triplicate determinations.

Supplementary Figure 2


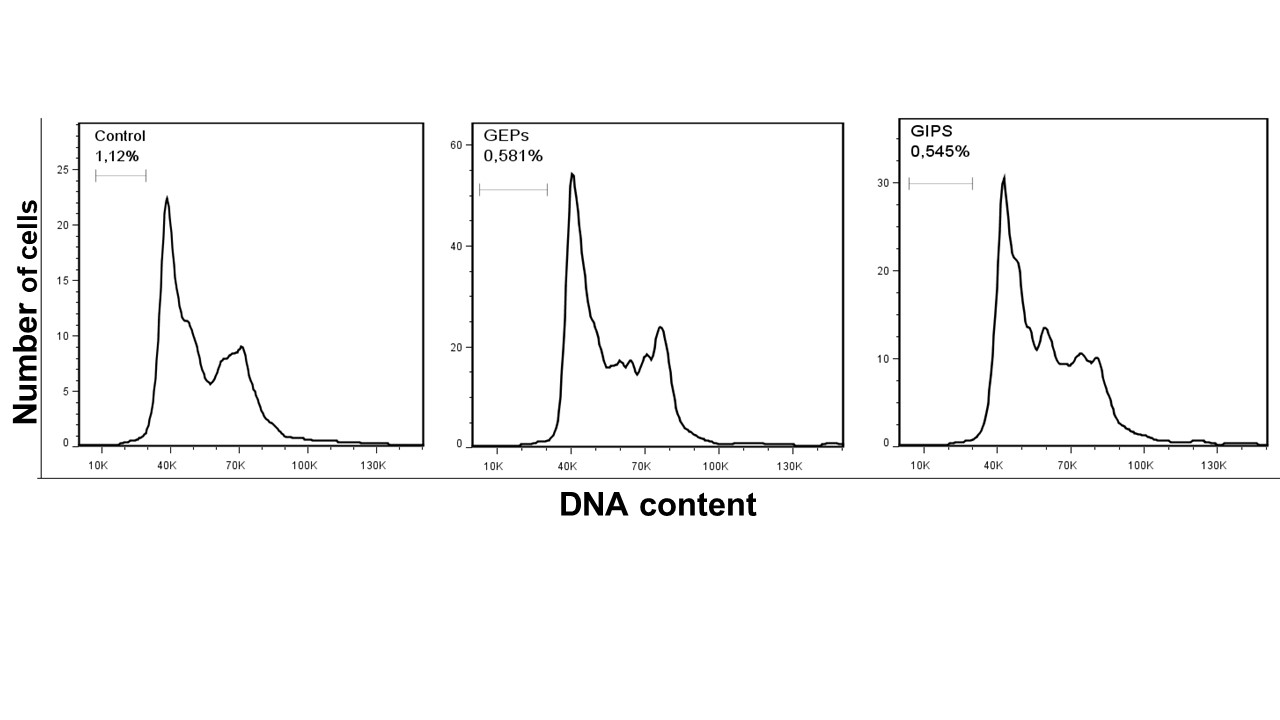
A


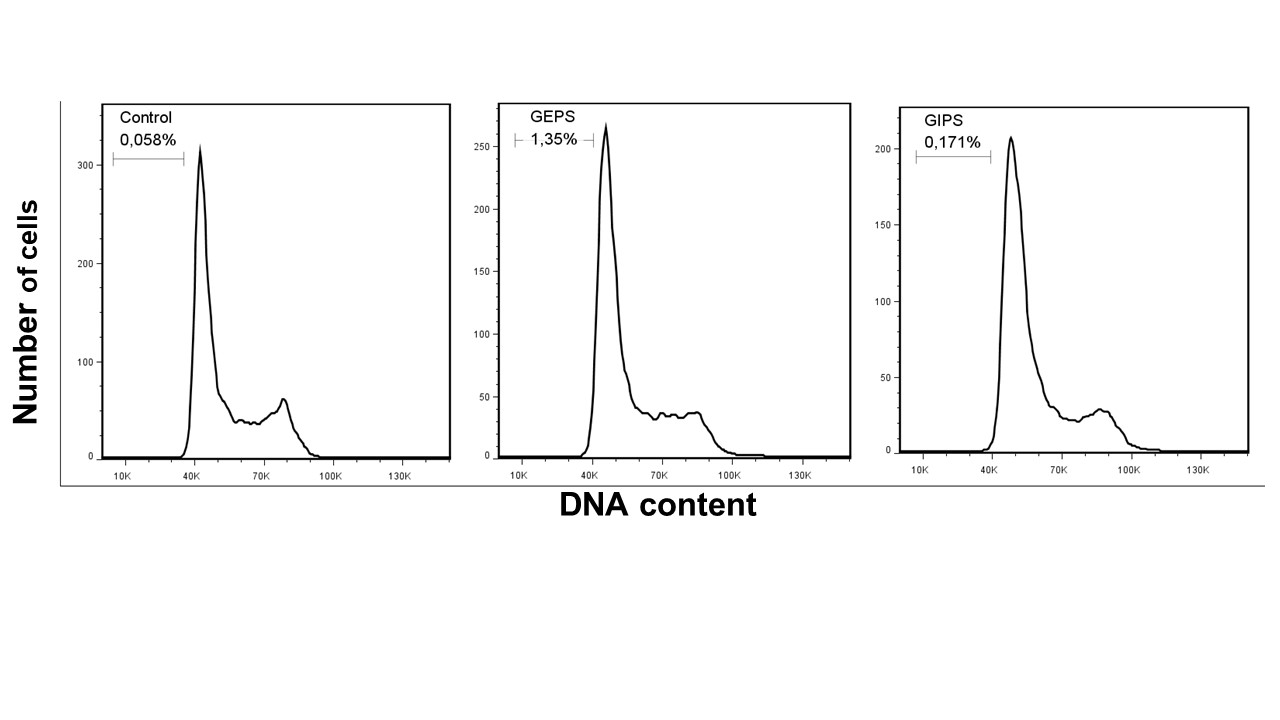
**B**

**Supplementary figure 2. PI-flow cytometric analysis of cell cycle.** Histograms present cell cycle distribution in cells LLC (A) and A549 (B) treated with GEPS and GIPS 48 h. Apoptosis cell death was quantitated as a percentage of hypodiploid cells (sub G0/G1).

Suplemmentary Figure 3

A

**Suplemmentary figure 3. Establishment of the tumor-associated macrophage model TAMs-Like.** Raw 264.7 macrophages were treated with IL4+ CoMe from LLC cells for 24 hours. Subsequently, the transcription of mRNA genes (TNF-a, IL-1B, IL-6, iNOS, Mrc1, Arg1 and Fizzi) was measured by qRT. -PCR.
